# Development of a sensitive and specific qPCR assay in conjunction with propidium monoazide for enhanced detection of live *Salmonella* spp. in food

**DOI:** 10.1186/1471-2180-13-273

**Published:** 2013-12-01

**Authors:** Baoguang Li, Jin-Qiang Chen

**Affiliations:** 1 Division of Molecular Biology, Center for Food Safety and Applied Nutrition, U.S. Food and Drug Administration, Laurel, MD, 20708, USA

**Keywords:** *Salmonella*, qPCR, Propidium monoazide, Live cells, *invA* gene

## Abstract

**Background:**

Although a variety of methodologies are available for detection of *Salmonella,* sensitive, specific, and efficient methods are urgently needed for differentiation of live *Salmonella* cells from dead cells in food and environmental samples. Propidium monoazide (PMA) can preferentially penetrate the compromised membranes of dead cells and inhibit their DNA amplification, however, such inhibition has been reported to be incomplete by some studies. In the present study, we report an efficient qPCR assay targeting a conserved region of the *invA* gene of *Salmonella* in conjunction with PMA treatment for detection of DNA from live *Salmonella* cells in food samples.

**Results:**

We investigated the relationship between amplicon length and inhibitory effect of PMA treatment to prevent DNA amplification from dead cells while allowing for DNA amplification from live cells, and found that the two factors are well correlated with each other. An amplicon that is 130 bp in length was determined to be optimal for PMA treatment and was selected for further PMA-qPCR assay development. A PMA-qPCR assay was established by utilizing this amplicon and adopting a modified PMA-treatment procedure. The PMA-qPCR assay provided excellent inhibition of DNA amplification from dead cells (a 17-*C*_*T*_-value, or 128,000-fold reduction) while only a slight DNA amplification difference (0.5 *C*_*T*_ value) was noted between the PMA-treated and untreated live cells. This assay has been validated through stringent inclusivity and exclusivity studies using a large number of (n = 167) *Salmonella*, including all strains of SARA and SARB collections, and non-*Salmonella* strains (n = 36). This PMA-qPCR assay is capable of detecting live *Salmonella* cells in live/dead cell mixtures, or 30 CFU/g live *Salmonella* cells from enriched spiked spinach samples as early as 4 h.

**Conclusions:**

A 130-bp amplicon in *invA* gene was demonstrated to be optimal for PMA treatment for selective detection of live *Salmonella* cells by PCR. This PMA-qPCR assay provides a sensitive, specific, and efficient method for detecting live *Salmonella* cells in foods and environmental samples and may have an impact on the accurate microbiological monitoring of *Salmonella* in foods and environment samples.

## Background

*Salmonella* is one of the most common foodborne pathogens, which causes diseases in humans, animals, and poultry worldwide [1,2]. It has been estimated that in the United States alone, *Salmonella* infection causes 1.4 million foodborne illnesses per year, which accounts for approximately 30% of total outbreaks and outbreak-related cases [1-3]. Furthermore, *Salmonella* infection has not declined significantly in more than a decade, resulting in an estimated $365 million in direct medical cost annually [4]. *Salmonella* infections in humans have been linked to a wide variety of sources such as under-cooked meats [5-7] and fresh produce [8,9]. Therefore, development of rapid, sensitive, and accurate methodologies for the detection of *Salmonella* in foods and environmental samples may have an impact on the public health burden caused by this foodborne pathogen.

Traditional methods for isolating and identifying *Salmonella* in food rely on nonselective and selective pre-enrichment, followed by isolation using selective and differential media. Isolated colonies are identified biochemically and by using serology [10]. The major limitation of these methods is that they typically take 4–8 days to obtain results. In addition, the sensitivity of the culture method, which is currently considered the gold standard for detection of *Salmonella*, is lower compared with that of DNA-based methods. This limitation may result in an increased false-negative rate [10,11]. To shorten detection time and reduce tedious work to perform traditional culture methods, immunoassays such as enzyme-linked immunosorbent assay (ELISA) have been used for detection of *Salmonella*[10,12], but poor performance in sensitivity and specificity as compared with other methods has relegated these methods to be a less than an ideal option for the field work [13]. Therefore, there is a need to develop rapid, sensitive and specific methodologies to detect this pathogen in foods*.* Recently, DNA-based molecular detection tools such as conventional and qPCR have been used for bacterial diagnostics [11,13-15]. More recently, qPCR is gaining popularity for its sensitivity, specificity, and rapid turnaround time. However, the use of these methods is hampered by their inability to distinguish DNA signals originated from live or dead cells. Because detection of live cells is most relevant in molecular diagnostics [16], it is essential to have reliable methods for selective detection of DNA from live *Salmonella* cells. To differentiate live and dead cells, several strategies have been used in molecular detection; one of the most commonly used strategies is to detect the presence of RNA which is inherently unstable [9,17,18]. However, it is known that working with RNA is cumbersome due to the risk of contamination with RNases and, hence can be labor intensive. Recent development of a photoreactive binding dye, propidium monoazide (PMA) offers an alternative way to differentiate dead cells from live cells [17,19,20] and has been successfully used for selective detection of live *Escherichia coli* O157H:7 cells from food by our group [21]. PMA is capable of penetrating membrane-compromised dead cells, but not intact live cells. Once the dye enters a cell, it can bind to DNA and covalently cross-link to the DNA upon light-exposure. Consequently, the amplification of such modified DNA is inhibited. However, in some cases, such inhibition of amplification of DNA of dead cells was found incomplete by several research groups [22-25].

Considering these factors, the present study embraced two objectives: first, we developed and evaluated a qPCR assay that not only improves sensitivity and specificity for detection of *Salmonella* but also is compatible in PMA-mediated inhibition of DNA amplification from dead cells; second, we developed a PMA-qPCR assay by combining the qPCR assay with PMA-treatment for selective detection of DNA from live cells from dead cells. Furthermore, we applied this assay for the selective detection of DNA from live *Salmonella* cells in spiked spinach and beef.

## Results

### Effect of amplicon length on inhibition of amplification of DNA from dead cells

In order to investigate whether PMA-mediated inhibition of DNA amplification from dead cells had any correlations with amplicon length, we designed five primer pairs that gave amplicons of five different lengths and made the comparison on their effects on DNA amplification. Primer pairs A, B, C, D, and E yielded amplicons of 65, 97, 119, 130, and 260 bp in length, respectively, and achieved *C*_*T*_ value differences 6.06, 11.55, 12.84, 13.18, and 15.44, respectively between the treated and untreated dead cells (Table 1). The results demonstrated that the PMA-mediated inhibition of DNA amplification of dead cells is well correlated to the amplicon length. On the other hand, when the amplicon length increased, the DNA amplification efficiency of the untreated dead cells decreased slightly except that the amplicon D (*C*_*T*_ value of 31.52) was slightly more efficient than that for amplicon C (*C*_*T*_ value of 33.38). Ultimately, amplicon D was selected for the further PMA-qPCR assay development based on its performance in inhibiting `sustaining DNA amplification from the treated or untreated dead cells, respectively (Table 1).

**Table 1:** **Effect of amplicon length on PMA-mediated inhibition of DNA amplification from dead cells in qPCR targeting* invA*** gene^**a**^

**Amplicon**	**Sequence of primers or probe**	**Position**	**Amplicon length (bp)**	***C***_***T ***_**value with PMA**	***C***_***T ***_**value w/o PMA**	***C***_***T ***_**value difference**^**b**^
	Forward 5′-CGTTTCCTGCGGTACTGTTAATT^c^	197-219				
	Probe FAM-CCACGCTCTTTCGMGBNFQ^d^	221-233				
A	Reverse 5′-ACGACTGGTACTGATGATCGATAATGC	261-238	65	23.81	17.75	6.06
B	Reverse 5′-ATTTCACGGCATCGGCTTCAATC	293-270	97	29.96	18.41	11.55
C	Reverse 5′-GAATTGCCCGAACGTGGCGATAAAT	315-292	119	33.38	20.54	12.84
D	Reverse 5′-TCGCCAATAACGAATTGCCCGAAC	326-303	130	31.52	18.34	13.18
E	Reverse 5′-TCGCCAATAACGAATTGCCCGAAC	456-435	260	35.53	21.19	15.44

^a^*invA* gene sequence is from GenBank accession number M90846.

^b^*C*_*T*_ value of untreated dead cells minuses *C*_*T*_ value of PMA-treated dead cells.

^c^The forward primer is shared by five reverse primers.

^d^The probe is shared by five primer pairs.

### Sensitivity of the qPCR assay

**Figure 1:**
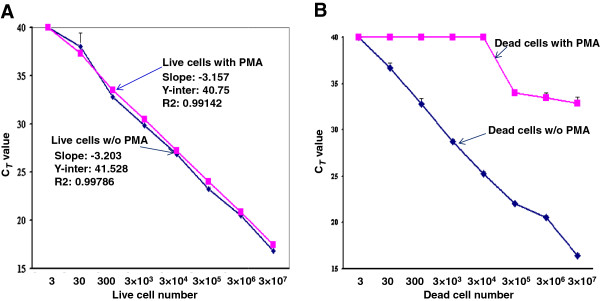
**Standard curves for detection of *Salmonella *by PMA-qPCR.** Live *Salmonella* cells treated with PMA or without PMA **(A)**; dead *Salmonella* cells treated with PMA or without PMA **(B)**. Results were the average of three independent assays with triplicates ± standard deviation.

The sensitivity studies of the qPCR assay developed in this study was performed using serial 10-fold dilutions of live and dead *Salmonella cells*. The standard curve established by the qPCR assay demonstrated with robust amplification efficiency, i.e., 105.21% for qPCR assay without PMA treatment, and 107. 375% for qPCR assay with PMA treatment. The detection limit of the assay was as low as 3 CFU (Figure 1A). In addition, we compared the live cells treated with PMA or without PMA side by side with standard curves in qPCR. The *C*_*T*_ values from the PMA-treated live cells (pink curve) and the untreated live cells (blue curve) appeared to be linear and nearly identical to each other. Only slight differences in *C*_*T*_ value (about 0.5) were seen between the PMA-treated and untreated live cells. These results indicated that PMA treatment did not significantly affect the amplification of DNA of live cells in the qPCR (Figure 1A). Most importantly, the amplification of DNA of dead cells was almost completely inhibited as shown in Figure 1B. The inhibition efficacy of DNA from dead cells was as high as 100% at a cell concentration of 10^4^ CFU/ml. At higher cell concentrations, PMA showed slightly less complete inhibitory efficacy with *C*_*T*_ values around 35. But with cell concentration increased to 10^7^ CFU/ml, the *C*_*T*_ value difference between the dead cells treated with PMA and without PMA reached 17 or 128,000-fold as shown in Figure 1B.

### Exclusivity and inclusivity of the qPCR assay

The specificity of the assay developed in this study was assessed by designing inclusive and exclusive studies. A large number of *Salmonella* strains, *E. coli* O157:H7, non-O157 STEC and *Shigella* strains were examined. The results indicated that all the *Salmonella* strains (n =167) were positively identified, and no cross-activity was detected with 36 *E. coli* O157:H7, non-O157 STEC strains, *Shigella* or other foodborne pathogens strains tested (Additional file 1: Table S1; Table 2).

**Table 2:** **Bacterial strains used in this study**^a^

**Group/genus and species**		**Strain name and serotype**		**No. strains**	**Year**^**d**^
*Salmonella* (n = 24)		SL856-874	Typhimurium	19	2009
		SL535	Typhimurium	1	2005
		SL302	Newport	1	2003
		SL317	Newport	1	2003
		SL192	Typhi	1	1996
Non-*Salmonella* strain (n = 36)					
*E. coli*		EC1275	O157:H7	1	
		EC118	ETEC	1	
		EC1434	DEC5A	1	
		EC1495	STEC	1	
		EC1472	STEC	1	
		EC1670	O121:HNM	1	
		EC1770	O26	1	
		EC1783	O111	1	
		EC1790	O145	1	
		EC1794	O145:NM	1	
		EC1795	O145:NM	1	
		EC1395	O26:H2	1	
		EC1403	O26:H-	1	
		EC1405	O111:H8	1	
		EC1406	O121:HNM	1	
		EC1407	O26:NM	1	
		EC118	ETEC	1	
		EC1434	DEC5A	1	
		EC1495	STEC	1	
		EC1472	STEC	1	
*Shigella*	*sonnei*	SH20145		1	
	*dysenteriae*	SH20152		1	
	*flexneri*	SH20155		1	
	*boydii*	SH20140		1	
*Klebsiella*	*pneumoniae*	ATCC13883		1	
*Pseudomonas*	*aeruginosa*	ATCC27853		1	
*Staphylococcus*	*aureus*	ATCC25923		1	
	*epidermidis*	ATCC12228		1	
	*pyogenes*	ATCC19615		1	
*Vibrio*	*alginolytica*	ATCC17749		1	
	*parahemolyticus*	ATCC17802		1	
	*vulasfians*	ATCC27562		1	
*Enterobacter*	*cloacae*	ATTCC23355		1	
	*cloacae*	ATCC13047		1	
	*cloacae*	ATCC13048		1	
*Citrobacter*	*freundii*	ATCC8090		1	

^a^*Salmonella enterica* strains (n = 144) of the SARA and SARB reference collections used in this study can be located in Additional file 1: Table S1).

^b^Strains from recent *Salmonella* outbreaks.

### Differentiation of live cells from live/dead cell mixtures

**Figure 2:**
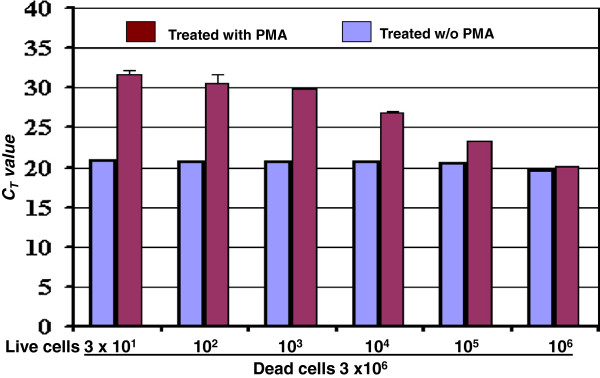
**Discrimination of live *Salmonella *cells from live/dead cell mixtures.** Dead cells at concentration of 3 × 10^6^ CFU/g were mixed with different number of live cells as indicated and treated with PMA or without PMA. Results were the average of three independent assays with triplicates ± standard deviation.

A set of 10-fold dilutions of live cells ranging from 3 × 10^1^ to 3 × 10^6^ CFU was treated with PMA or without PMA to differentiate live cells from dead cells. A progressive trend in *C*_*T*_ values that was in a reciprocal relationship with the live cell numbers in the cell mixtures was observed in Figure 2 (purple bars). This downward trend in *C*_*T*_ values was in a reciprocal relationship with the real number of live cells in the mixtures in spite of the presence of a large number of dead cells. These data demonstrated that the *C*_*T*_ values on the cell mixtures preferentially reflected the amount of DNA of the live cells in the mixtures amplified during the qPCR reaction. In contrast, the *C*_*T*_ values of the untreated cell mixtures were close together and failed to reflect the real number of live cells in the cell mixtures in Figure 2 (blue bars).

### Detection of live salmonella cells from spiked spinach and beef

**Figure 3:**
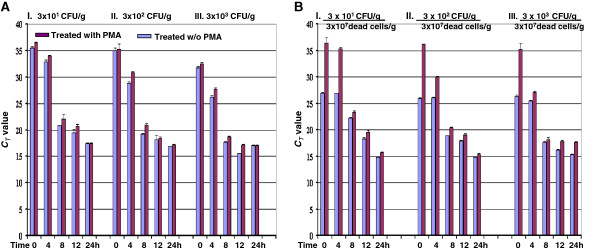
**Detection of live *Salmonella *cells spiked in spinach by PMA qPCR.** Spinach samples were inoculated with 3 × 10^1^ CFU/g, 3 × 10^2^ CFU/g and 3 × 10^3^ CFU/g of live cells, respectively **(A)**; spinach samples were inoculated 3 × 10^7^ dead cells/g and with 3 × 10^1^ CFU/g, 3 × 10^2^ CFU/g, and 3 × 10^3^ CFU/g of live cells, respectively, as indicated **(B)**. Spinach samples were incubated at 35°C up to 24 h. Incubated samples were collected at different time points and treated with PMA or without PMA before DNA extraction. Results were the average of triplicates ± standard deviation.

The PMA-qPCR assay was applied to detect DNA from live *Salmonella* cells in spiked spinach samples. The results showed that the *C*_*T*_ values of spinach samples were reversely correlated with the inoculated *Salmonella* live cell numbers and duration of enrichment (Figure 3A). Samples inoculated with 3 × 10^1^ and 3 × 10^2^ CFU/g of cells and without (0-h) enrichment yielded *C*_*T*_ values >35 either with PMA treatment or without PMA treatment (0-h), which were generally considered as negative results for qPCR. However, the sample inoculated with 3 × 10^3^ CFU/g of cells at 0-h enrichment was positive for *Salmonella* with C_*T*_ values of 32.48 and 31.74 with or without PMA treatment. The samples with 3 × 10^1^, 3 × 10^2^, and 3 × 10^3^ CFU/g of cells at 4-h enrichment were positive for *Salmonella* with *C*_*T*_ values of 33.98, 30.89, and 27.71 with PMA treatment and 32.91, 28.84, and 26.71 without PMA treatment, respectively. Samples with any concentrations (3 × 10^1^-10^3^ CFU/g) of *Salmonella* cells at 8-h or longer enrichment were positive for *Salmonella* either with or without PMA treatment (Figure 3A).

We further tested the PMA-qPCR assay for detection of DNA from live *Salmonella* cells in the presence of a large number of dead cells from spiked spinach samples (Figure 3B). The samples inoculated with 3 × 10^1^, 3 × 10^2^, and 3 × 10^3^ CFU/g of cells without (0-h) enrichment generated *C*_*T*_ values of 25.94, 26.89, and 26.29 without PMA treatment but three samples after PMA treatment yielded *C*_*T*_ values all >35, indicating that the positive readings were due to the presence of a large number of dead cells. With 4-h enrichment, the sample with 3 × 10^2^ CFU/g of cells was positive for *Salmonella* with *C*_*T*_ values of 29.85 or 26.89 with or without PMA treatment (Figure 3B II). Similar trends were found in the samples inoculated with 3 × 10^3^ (Figure 3B I), 3 × 10^1^ (Figure 3B III). A downward trend in *C*_*T*_ values was seen as a function of time. These results indicated the incapability of PCR alone to differentiate DNA from live and dead cells and the necessity for PMA treatment before DNA extraction.

Similar results were obtained with spiked beef samples. The beef samples inoculated with 30 CFU/g of cells were detected *Salmonella* after 4-h enrichment with *C*_*T*_ values of 32.81. (Additional file 2: Table S2). Together, these results confirmed that this PMA-qPCR assay selectively detected 30 CFU/g live *Salmonella* cells from spiked spinach samples after 4-h enrichment (Figure 3B).

## Discussion

In spite of the fact that there are numerous DNA-based molecular methods available for detection of *Salmonella*, there is still room for improvement in qPCR assays to detect live *Salmonella* cells from foods and environment samples. To our knowledge, this is a first new qPCR assay for selectively detect live *Salmonella* cells that has been validated with such a comprehensive coverage of the *Salmonella* group, including strains of SARA (n = 72) and SARB (n = 72) collections and strains of recent outbreaks (n = 23). Furthermore, this assay is highly sensitive and specific for the detection of live *Salmonella* cells, and PMA-treatment is able to efficiently inhibit the DNA amplification from dead cells but has little effect on the DNA amplification from live cells.

**Figure 4:**
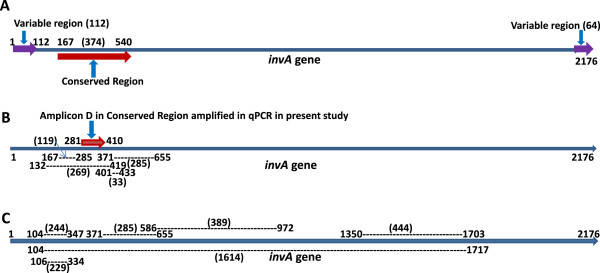
**Heterogenic sequences in *invA *gene demonstrated among *Salmonella *strains by BLAST.** It is more intensive at the 5′- and 3-′ ends **(A)**. Target regions (or amplicons) in *invA* gene used for detection of *Salmonella* by PCR from previous reports were indicated with dash lines. Numbers in the *invA* gene are nucleotide positions of the 5′- or 3-′ ends of the amplicons in PCR detection schemes (see references in Table 3), and numbers in parentheses represent amplicon length in bp in qPCR assays **(B)** and conventional PCR assays **(C)**. Subjects in the figure are not in scale.

We chose the *invA* gene, the invasive gene *in Salmonella,* as a target gene in the qPCR assay for several reasons: first, the *invA* gene is an important virulence factor gene [26] and is considered present in all *Salmonella* spp. [27,28]; second, currently, most molecular-based assays for the detection of *Salmonella* are *invA-*based, especially for conventional PCR and qPCR assays; and third, the *invA-*based PCR assays have demonstrated inclusivity for a wide range of *Salmonella* serotypes including all subspecies and exclusivity for other closely related species and genera [29]. In general, the *invA-*based PCR assays provide higher sensitivity, shorter turnaround time, and reduced labor cost, making it an excellent alternative to conventional culture method for pathogen detection [29]. However, conspicuous variations in sensitivity and specificity of *invA-*based PCR assays have been documented by numerous studies [1,29-35], and one of the possible reasons for such discordant outcomes may be due to the use of different primers for gene detection in the assays such as conventional or qPCR [36]. In an effort to better understand the variations caused by the usage of different primers for gene detection in PCR assays, we systematically evaluated the most commonly used *invA* primer pairs for the detection of *Salmonella* in thirteen (n = 13) PCR assays (Table 3; Figure 4). First, although the *invA*-based PCR assays generate reasonably good results for *Salmonella* detection, in some cases, the false-negative and false-positive rates were rather high [29]. The reasons for these false-negative and false-positive results are not clear, but primers and probes used for gene detection may be to blame. Although the *invA* gene is encoded by almost all strains in *Salmonella* spp. examined, our BLAST sequence analysis revealed that the *invA* gene sequence is rather heterogenic among the *Salmonella* group of more than 2600 serotypes, especially at the 5-′ and 3′- ends of the gene. Furthermore, regions further into the gene, single nucleotide polymorphisms (SNPs) occur sporadically at different locations with variable frequencies among *Salmonella* spp. Inevitably, it becomes a formidable task to detect such a broad and diversified *Salmonella* group by targeting a single gene. If previously designed primer pairs listed in Table 3 are used, several PCR assays would fail to detect numerous *Salmonella* spp., whose sequences are currently available in GenBank. This could partially explain the false-negative results encountered in *Salmonella* detection [36]. At the same time, although *invA* is capable of excluding non-*Salmonella* strains, our BLAST sequence analysis of *invA* demonstrated that some non-*Salmonella* groups such as *E. coli, Staphylococcus* aureus subsp. aureus, and *Solanum lycopersicoides* shared identities with *Salmonella invA.* This could give a possible explanation for the false-positive results reported by some analysis [36].

**Table 3:** **PCR primer pairs used for targeting *invA *** **gene for detection of *Salmonella***

**Primer sequence (5′---3′)**	**Type of PCR**	**Position**	**Length (bp)**	**Reference (year)**
GCTGCGCGCGAACGGCGAAG	Conventional	586-608	389	Ferretti et al. (2001)
TCCCGGCAGAGTTCCCAT T		972-954		
ACAGTGCTCGTTTACGACCT AAT	Conventional	104-127	244	Chiu and Ou (1996)
AGACGACTGGTACTGATCGATAAT		347-324		
GTGAAATAATCGCCACGTTCGGGCAA	Conventional	371-396	285	Malorny and Hoorfar (2005)
TCATCGCACCGTCAAAGGAACC		655-634		
GTGAAATAATCGCCACGTTCGGGCAA	Conventional	371-396	285	Rahn et al. (1992) [28]
TCATCGCACCGTCAAAGGAACC6		655-634		
AGTGCTCGTTTACGACCTGAA	Conventional	106-126	229	Mainar-Jaime et. al. ( 2013) [29]
TGATCGATAATGCCAGACGA		334-315		
ACAGTGCTCGTTTACGACC	Conventional	104-122	1614	Banihashemi et al. (2012) [31]
TACGCACGGAAACACGTTC		1717-1699		
TTTACGGTCTATTTTGATTTG	Conventional	1350-1370	444	Arnold et al. (2004) [30]
ATATGCTCCACAAGGTTAATG		1703-1683		
TTATTGGCGATAGCCTGG	Real-time	401-418	33	ABI, (1999)
CGGTGGGTTTTGTTG		433-419		
TTGGCGATAGCCTGGCGGTG	Real-time	404-423	136	Braun et al. (2011) [35]
TGTTTACCGGGCATACCATCCAGAG		539-515		
TCGTCATTCCATTACCTACC	Real-time	167-186	119	Hoorfar et al. (2000) [33]
AAACGTTGAAAAACTGAGGA		285-266		
GATTCTGGTACTAATGGTGATGATC	Real-time	132-156	269	Liang et al. (2011) [34]
GCCAGGCTATCGCCAATAAC		419-400		
GTGAAATAATCGCCACGTTCGGGCAA	Real-time	371-396	285	Chen et al. (2011) [32]
TCATCGCACCGTCAAAGGAACC		655-634		
CGTTTCCTGCGGTACTGTTAATT	Real-time	281-303	130	This study
TCGCCAATAACGAATTGCCCGAAC		410-387		

**Figure 5:**
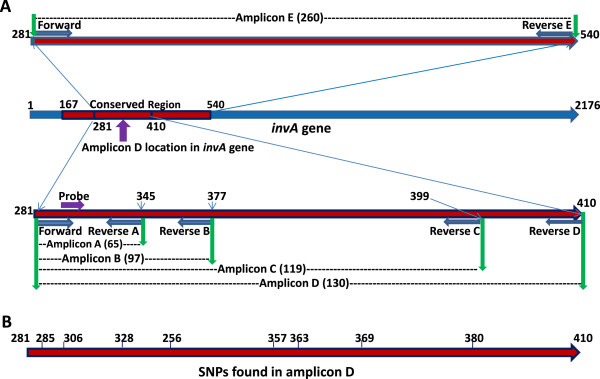
**The strategy used for the development of PMA-qPCR assay for detection of *Salmonella.*** Five primer pairs were designed in the conserved region near the 5′-end of *invA* gene (red block, from nucleotide positions 167 to 540). All five primer pairs shared the same forward primer and probe, and the reverse primers (A, B, C, D, and E) defined the amplicon length of amplicons A through E (Figure 5**A**); the numbers on amplicon D represent the locations of most of the SNPs found between the sequence of amplicon D in *invA* gene of *Salmonella* Typhimurium and the available *invA* gene sequences in GenBank. The number in parentheses indicates the amplicon length in bp (Figure 5**B**). Subjects in the figure are not in scale.

Fortunately, with the usage of new high throughput sequencing platforms, many genomic sequences, including *Salmonella* spp., are available to the public. It has become more feasible to find specific sequences within *invA* gene that are highly conserved among *Salmonella* spp. that can be used as specific genetic markers for *Salmonella* spp. to detect many more *Salmonella* serotypes. With BLAST analysis of the *invA* gene sequence of *Salmonella* Typhimurium, we found a highly conserved segment of sequence (374 bp) near the 5′-end of the *invA* gene (Figure 4A), which several *invA*-based PCR assays have been used to target part of or the whole segment (Figure 4B;C). We took advantage of this characteristic of the *invA* gene to design five primer pairs in that region (Figure 5A). To enhance PMA-mediated inhibition of DNA amplification from dead cells, primer pairs were selected for one that generated high efficacy in inhibition of DNA amplification from dead cells and provided robust efficiency in DNA amplification from live cells as well. Another parameter we took into account was the compatibility between the PMA-treatment and qPCR efficiency. One study found that efficient PMA-mediated inhibition of DNA amplification required amplicons at least 190 bp in length [23]. This can be achieved when conventional PCR is in use, but amplicons longer than 190 bp might not work well in qPCR as shown in Table 1. Subsequently, an optimal amplicon (D) size of 130 bp was determined and selected for the qPCR assay development through numerous trials where PCR parameters and PMA-treatments were varied (Table 1). With amplicon D, this qPCR assay offers high sensitivity (Figure 1), and has been validated with a large number of *Salmonella* strains (n = 167), covering all strains from SARA (n = 72), SARB collections (n = 72) and collection strains from the recent *Salmonella* outbreaks (n = 23) (Additional file 1: Table S1; Table 2). All the *Salmonella* strains examined were positively identified without exception. This qPCR assay delivers low background on non-*Salmonella* strains, such as *E. coli* O157:H7, STEC, *Shigella,* or other foodborne pathogens (Table 2). The excellent performance in sensitivity and specificity is not a surprise; rather there are underlining reasons: (a) BLAST analysis of the sequence of amplicon D demonstrated that this fragment shares a remarkably high homology with most of the currently available *invA* sequences of *Salmonella* spp*.* It showed 100% identity with 16 genomic sequences, 99% identity (1 SNP) with 26 sequences, 98% of identity (2 SNPs) with 9 sequences, and 97% or lower identity with other sequences. (b) The positions of the mismatches with other *Salmonella* strains are illustrated in Figure 5B. Of the strains that showed mismatches, at least 5 strains belong to *Salmonella* bongori subgroup. More importantly, most of the mismatches were not located in the sequences targeted by the primers and probe we used, therefore, the changes would not affect the inclusivity of the PCR assay strategy. In contrast, numerous mismatches were found between the previously designed primer pairs listed in Table 3 and the published *invA* sequences of *Salmonella*. (c) Furthermore, we have applied this qPCR assay for detection of *Salmonella* from environmental water samples, which were collected and shipped to DMB lab from irrigation ponds in vegetable growing farms in southern Georgia, USA. Briefly, the water samples were concentrated by filtration, enriched with LB broth at 37°C for 24 h, purified for DNA, and subjected to this qPCR assay for detection of *Salmonella*. Of 150 water samples tested, over forty have been positive for *Salmonella* by this qPCR assay (Li et al. 2013 ASM Abstract). More significantly, we have isolated a *Salmonella* strain by standard culture method (FDA BAM) from every qPCR-positive (*C*_*T*_ value under 35) water sample; and every *Salmonella* isolate was subsequently confirmed by traditional identification methods, and genotyped by genotyping microarray. And thus, the successful application of this qPCR assay for detection of *Salmonella* from irrigation water samples is testimonial for the high sensitivity and specificity of the qPCR assay (Li et al. 2013 ASM Abstract).

Our second objective was to remedy a drawback of PCR’s inability to distinguish signals originated from live or dead cells, by combining the qPCR with PMA treatment. *Recently*, PMA has been used for differentiation of live cells in qPCR [16,19-21,24,32,34,37,38] However, several studies revealed that the inhibition of amplification of DNA of dead cells was incomplete [22,23,37,39]. In order to improve the efficacy of PMA treatment, we evaluated the effect of amplicon length on PMA-mediated inhibition of DNA amplification from dead cells by qPCR (Table 1). We found efficacy of PMA treatment appeared to be well correlated to the amplicon length, which is in good agreement with the previous finding [23]. However, our results showed significant differences with their conclusion on efficiency of amplicon length, i.e. PMA-mediated suppression of DNA amplification from dead cells was incomplete with amplicons shorter than 190 bp [23]. With amplicon D (130 bp), we were able to achieve a *C*_*T*_ value difference of 13.1 between the treated and untreated dead cells (Table 1). Although amplicon E (260 bp) generated a bigger *C*_*T*_ value difference (15.44), the C_*T*_ value for DNA of untreated dead cells increased from 18.34 to 21.19, reflecting about a 3-*C*_*T*_-value decrease in sensitivity of the PMA-qPCR assay (Table 1). This finding is of importance because it can give guidance for selection of primer pairs for the development of qPMA-PCR assays. There are no good theoretical explanations for this “amplicon length effect” associated with PMA treatment. It may be related to the mechanism of the PMA-treatment. When dead cells are treated with PMA, the DNA is blocked by covalent bonds and thus it cannot be amplified in PCR [38]. It could be understood that the larger an amplicon is, the longer the region that the polymerase needs to cover, the higher probability for the target DNA being blocked by a covalent bond (s). On the other hand, if the amplicon length is too long (over 200 bp), the sensitivity of the qPCR will be compromised, resulting in lower sensitivity of the assay. This finding has significance to future designs of qPCR assay in general.

Consumption of fresh produce including salads, lettuce, juice, melon, sprouts, and berries has been identified as important sources for *Salmonella* outbreaks [40]. It is important to accurately monitor live cells in food samples, because only live bacteria can cause disease [16]. We applied PMA-qPCR technology to selectively detect low numbers of live *Salmonella* cells in spiked spinach samples. This PMA-qPCR assay positively detected *Salmonella* in spinach spiked with 30 CFU/g at 4-h enrichment or from samples inoculated with 3 **×** 10^3^ CFU/g without enrichment (Figure 3A). Additionally, with this PMA-qPCR assay, we were able to detect 30 CFU/g live cells with a 4-h enrichment in the presence of large number of dead *Salmonella* cells (3 **×** 10^7^/g) (Figure 3B). This is an improvement in sensitivity compared with recent reports on detection of *Salmonella*. Live *Salmonella* cells were detected from spiked lettuce samples at the concentration of 10^1^ CFU/g with 12-h enrichment [34]. Another study reported that the detection limit of PMA-LAMP (loop-mediated isothermal amplification) was 6.1 **×** 10^3^-10^4^ CFU/g in spiked produce and PMA-PCR was up to 100-fold less sensitive compared with qPCR assay [32]. It is noteworthy to mention that this PMA-qPCR assay reported here appears to be more sensitive. Two factors might explain this: first, it may be due to the qPCR assay we developed in this study, which offers higher sensitivity with detection limit as low as 3 CFU; whereas the two previous assays used longer amplicons (269 bp and 285 bp) in their qPCR assays [32,34], which would make the qPCR assay less efficient compared with the assays with shorter amplicons; second, it might be due to the usage of our previously modified PMA-treatment procedure, which was shown to increase the PMA-qPCR efficiency [21]. With this modified PMA-treatment procedure, not only could we achieve a relatively small *C*_*T*_ value difference (0.5) between treated and untreated live cells (Figure 1A), but we were also able to obtain efficient inhibition (17-*C*_*T*_-value difference, 128,000-fold) of DNA amplification with dead cells (Figure 1B). These improvements made it possible for efficient and accurate differentiation of live *Salmonella* cells from dead cells by this PMA-qPCR assay [37]. Furthermore, we have successfully applied this assay to detect live *Salmonella* cells from beef (Additional file 2: Table S2) and environmental water samples [41]. It may be applied to other food matrices as well, fostering improvement of accurate monitoring *Salmonella.*

## Conclusions

We have developed a PMA-qPCR assay for selective detection of live *Salmonella* cells from dead cells in food. This assay is sensitive and specific and has been validated with a large number of *Salmonella* strains. We were able to differentiate live *Salmonella* cells from live/dead cell mixtures. This PMA-qPCR has been applied for selective detection of live *Salmonella* cells in spiked spinach. It allows selective detection of 30 CFU/g *Salmonella* from spiked spinach with 4-h enrichment. Additionally, we evaluated the effect of amplicon length on PMA-mediated inhibition of DNA amplification of dead cells. The limitation of this PMA-qPCR assay is that PMA treatment slightly increases the cost and reduces the sensitivity of PCR assay.

## Methods

### Bacterial strains

*Salmonella* Enteritidis (SARB16) was used in designed experiments of optimization, sensitivity, and spinach spiking. *Salmonella* strains used for inclusive and exclusive evaluations included all strains from the *Salmonella* Reference A (SARA) (n = 72) [42] and *Salmonella* Reference B (SARB) (n = 72) [43], strains from recent *Salmonella* outbreaks and internal strain collections (n = 23) of the Division of Molecular Biology (DMB), Food and Drug Administration (FDA), (Additional file 1: Table S1; Table 2). Additionally, numerous non-*Salmonella* strains (n = 36) were shown in Table 3 for exclusivity testing, including *E. coli* O157:H7, non-O157 Shiga toxin-producing *E. coli* (STEC) strains, *Shigella* and other foodborne pathogen strains.

### Bacterial growth

All bacteria were grown in Luria Bertani (LB) broth (Becton Dickinson and Company, Sparks, MD) at 37°C with shaking at 180 rpm, or as otherwise stated. Growth of *Salmonella* Enteritidis (SARB16) was monitored by determining the turbidity at 600 nm (OD_600_) using a DU530 spectrophotometer (Beckman, CA). To enumerate bacterial cells, cultures were diluted serially in 10-fold increments with LB medium and plated onto LB agar plates at 37°C overnight.

### DNA extraction

DNA was extracted from bacterial cultures using the Puregene cell and tissue kit (Gentra, Minneapolis, MN) according to the manufacturer’s instructions. Briefly, 1 ml of overnight grown culture was centrifuged, resuspended with 3 ml of cell lysate solution, and incubated at 80°C for 5 min. Fifteen microliters of RNase A solution was added, mixed, and incubated at 37°C for 60 min. One milliliter of protein precipitation solution was added, vortexed and centrifuged. The supernatant was combined with 3 ml of 2-propanol, mixed, and centrifuged. The pellets were washed with 70% ethanol, rehydrated with 500 l of DNA hydration solution, and incubated at 65°C for 1 h. The DNA concentrations were determined by measuring optical density (OD_260_) using a spectrophotometer (NanoDrop Technology, Wilmington, DE).

### Primers and probes

The sequence of the *invA* gene used in this study was identified from the genomic sequence of GenBank accession number M90846. Primers and probe were designed using Primer Express^©^ 3.0 software from Applied Biosystems Inc. (ABI, Foster City, CA). Five primer pairs that encode different lengths of amplicons were designed and are listed in Table 1.

### qPCR assay conditions

Reaction mixtures consisted of 12.5 μl of 2 × Universal Master Mix (ABI), 200 nM of forward and reverse primers targeting *invA* gene in *Salmonella* and 100 nM of probe. Template DNA (5 μl of 20 pg/μl) and an appropriate volume of nuclease-free water (Qiagen Sciences, MD) were added to reach a final reaction volume of 25 μl. qPCR conditions were set as follows: activation of TaqMan at 95°C for 10 min; followed by 40 cycles of denaturation at 95°C for 10 s and annealing/extension at 60°C for 1 min.

### qPCR with internal amplification control

To ensure the amplification was free of inhibitory factors from examined samples, an internal amplification control (IAC) was set. The primers and probe for IAC were designed [21,44] based on the pUC19 DNA (Promega, Madison, MI), which was diluted to 50 fg/μl. The sequences of primers and probe used in the study were as follows: IAC-Forward, 5′-CAGGATTGACAGAGCGAGGTATG; IAC-Reverse, 5′-CGTAGTTAGGCCACCACTTCAAG; and IAC-probe, VIC-AGGCGGTGCTACAGAG- MGBNFQ. For each reaction, 0.5 μl of IAC forward and reverse primers (100 μM), 0.25 μl of IAC-probe (10 μM), and 1 μl of diluted pUC19 DNA (1.8 × 10^4^ copies) were added to the regular qPCR reaction mixture components as described above to reach the final reaction volume of 25 μl. qPCR was performed using the same conditions as described above.

### Sensitivity test and detection limit of the qPCR assay

A *S*a*lmonella* Enteritidis (SARB16) culture was grown at 37°C to mid-exponential phase (OD_600_ = 0.5), and was divided into two aliquots. One aliquot was boiled for 10 min in a water bath to produce heat-killed cells; the other aliquot was used for live cells. The absence of live cells from the heat-killed cells was confirmed by plating the cells onto LB agar plates. The live and heat-killed aliquots were serially 10-fold diluted from 3 × 10° to 3 × 10^7^ CFU/ml with LB medium. Both the live and heat-killed cells suspensions were equally divided to make four sets of cell suspensions. One set of the live cell suspensions was treated with PMA and the other set was left untreated. Subsequently, standard curves were generated side by side for PMA-treated cells and untreated cells in the qPCR assay (Figure 1A). Likewise, PMA-treated or untreated dead cell suspensions were also subjected to qPCR analysis for generation of standard curves (Figure 1B).

### Inclusivity and exclusivity tests

A large number (n = 167) of *Salmonella* strains, including strain from FDA collections and recent outbreak isolates (Additional file 1: Table S1; Table 2), were used in inclusivity study. *Salmonella* strains from the SARA and SARB collections and other groups. *E. coli* O157:H7, non-O157 STEC strains, *Shigella*, and other pathogenic strains were used for exclusivity test (Table 2). DNA samples were prepared from the cultures of strains (Additional file 1: Table S1; Table 2) grown overnight at 37°C with a Wizard Plus Minipreps DNA Purification System Kit (Promega, Madison, WI). DNA concentration was adjusted to 20 pg/μl with water and 100 pg (5 μl) of DNA was used for the inclusivity and exclusivity studies in qPCR, and 5 μl of water was used as a no-template-control.

### Preparation of mixtures of live and dead cells for PMA-qPCR

*Salmonella* Enteriditis SARB 16, grown at 37°C to mid-exponential phase (OD_600_ = 0.5), was divided into two aliquots. One aliquot was boiled for 10 min in a water bath for heat-killed cells; the other was not boiled to represent corresponding live for live cells. The absence of live cells from the heat-killed cells was confirmed by plating the cells onto LB agar plates. Both the live and the heat-killed aliquots were diluted (10 fold) to 3 × 10^1^ to 3 × 10^7^ CFU/ml with LB medium and equally divided to make four sets of cell suspensions. The first two sets were used for cell mixtures of live and dead cells; one set was for the PMA-treated cells and the other was for the untreated cells. The third and fourth sets of cells were for PMA-treated live cell dilutions and untreated live cell dilutions.

### Combination of qPCR with PMA treatment

PMA treatment was performed as described earlier [21]. Briefly, separate live cells, heat-killed cells, and live/dead cell mixtures were aliquoted 100 μl in three 1.5-ml microtubes. Two microliters of 10 mM PMA was added to each aliquot to a final concentration of 50 μM. The samples were first incubated at room temperature in the dark for 5 min, with gentle shaking. Then the samples were exposed to a 650-W halogen light source, followed by DNA preparation, and qPCR analysis.

### Detection of live salmonella cells in spiked spinach and beef samples using PMA-qPCR

Fresh spinach and ground beef purchased from a local retail source, which were confirmed to be free of *Salmonella* by standard FDA BAM methods [45], was used for the spiking studies. The studies consisted of two parts. In part 1, three spinach samples (25 g) and three beef samples (25 g) were inoculated with 3 × 10^1^, 3 × 10^2^ and 3 × 10^3^ CFU/g *Salmonella* strain SARB16. In part 2, three samples three beef samples (25 g) were each inoculated with 3 × 10^7^/g dead cells and with 3 × 10^1^, 3 × 10^2^, and 3 × 10^3^ CFU/g of live cells, respectively. Each spinach or beef sample was mixed with 225 ml of LB medium and homogenized for 2 min using a stomacher (Seward, England). Five milliliters of the enriched cultures was collected at 0, 4, 8, 12 and 24 h after incubation at 37°C with shaking at 180 rpm. The collected samples were centrifuged at 600 × g for 1 min to collect leaf or fat tissues. The supernatants were transferred to 2.0-ml microtubes and centrifuged at 3000 × g for 5 min to collect cells. The cell pellets were suspended in 1.5 ml of LB medium and treated with PMA before DNA extraction and qPCR analysis.

## Abbreviations

CFU: Colony-forming units; PCR: Polymerase chain reaction; E. coli: *Escherichia coli*.

## Competing interests

The authors declare that they have no competing interests.

## Authors’ contributions

BL conceived and designed the study, performed experiments, and wrote the manuscript. J-QC performed experiments and participated in writing the manuscript. Both authors read and approved the final manuscript.

## Supplementary Material

Additional file 1: Table S1 *Salmonella enterica* strains of the SARA and SARB reference collections used in this study. Click here for file

Additional file 2: Table S2 Selective detecion of live *Salmonella* cells spiked in beef by PMA-qPCR. Click here for file
