# Supplementary material for: Development of a sensitive and specific qPCR assay in conjunction with propidium monoazide for enhanced detection of live Salmonella spp. in food
Source: BMC Microbiol. 2013 Dec 1;13:273. doi: 10.1186/1471-2180-13-273 (PMC4219372; doi:10.1186/1471-2180-13-273)
Supplement: Supplementary file 1 [file 1471-2180-13-273-S1.doc]

Table 2 ***Salmonella enterica*** **strains of the SARA and SARB reference collections used in this study**

Strain	Serovar	Host	Country	Year	Strain	Serovar	Host	Country	Year
SARA1	Typhimurium	Human	Mexico		SARB1	Agona		Peru	
SARA2	Typhimurium				SARB2	Anatum	Human	USA	
SARA3	Typhimurium	Horse	USA	1987	SARB3	Brandenburg		UK	1988
SARA4	Typhimurium	Rabbit	USA	1986	SARB4	Choleraesuis var. Kunzendorf	Pig	USA	1986
SARA5	Typhimurium		Mongolia		SARB5	Decatur		USA	
SARA6	Typhimurium	Human	USA		SARB6	Choleraesuis var. senso		Thailand	1954
SARA7	Typhimurium		Norway		SARB7	Decatur		Australia	1988
SARA8	Typhimurium		Finland		SARB8	Decatur		France	
SARA9	Typhimurium	Parrot	USA	1987	SARB9	Derby	Avian	USA	1986
SARA10	Typhimurium	Opossum	USA	1987	SARB10	Derby	Cow	USA	1986
SARA11	Typhimurium		Thailand		SARB11	Derby	Turkey	USA	1986
SARA12	Typhimurium	Horse	USA	1987	SARB12	Dublin	Cow	USA	1986
SARA13	Typhimurium		France		SARB13	Dublin	Cow	France	1982
SARA14	Typhimurium		Panama		SARB14	Dublin/Enteritidis	Human	Thailand	
SARA15	Typhimurium	Dog	USA	1987	SARB15	Duisburg		UK	1988
SARA16	Typhimurium	Human	USA		SARB16	Enteritidis		USA	
SARA17	Typhimurium		Yugoslavia		SARB17	Enteritidis	Human	USA	1982
SARA18	Typhimurium	Horse	USA	1987	SARB18	Enteritidis		USA	1988
SARA19	Typhimurium	Human	Mexico		SARB19	Enteritidis		Switzerland	1988
SARA20	Typhimurium		France		SARB20	Emek		Israel	
SARA21	Typhimurium	Heron	USA		SARB21	Gallinarum	Human	USA	1972
SARA22	Saintpaul	Human	USA		SARB22	Haifa		Scotland	1988
SARA23	Saintpaul	Human	USA		SARB23	Heidelberg	Chicken	USA	1987
SARA24	Saintpaul	uman	USA		SARB24	Heidelberg		Thailand	
SARA25	Saintpaul		France		SARB25	Indiana		UK	1988
SARA26	Saintpaul	Human	France	1988	SARB26	Infantis	Human	USA	
SARA27	Saintpaul	Human	France	1988	SARB27	Infantis		Senegal	
SARA28	Saintpaul	Human	France	1988	SARB28	Miami	Human	USA	1953
SARA29	Saintpaul	Human	USA		SARB29	Miami	Human	Guiana	1979
SARA30	Heidelberg	Chicken	USA	1987	SARB30	Montevideo	Human	USA	
SARA31	Heidelberg	Pig	USA	1987	SARB31	Montevideo	Human	USA	
SARA32	Heidelberg	Dog	USA	1986	SARB32	Muenchen			
SARA33	Heidelberg	Human	Mexico		SARB33	Muenchen	Human	France	1988
SARA34	Heidelberg		Israel		SARB34	Muenchen	Human	France	1988
SARA35	Heidelberg		Brazil		SARB35	Muenchen	Human	USA	

SARA37	Heidelberg	Turkey	USA	1987		SARB37	Newport	Human	Mexico	
SARA38	Heidelberg	Turkey	USA	1987		SARB38	Newport	Snake	USA	1987
SARA39	Heidelberg	Human	USA			SARB39	Panama		Italy	
SARA40	Heidelberg		USA			SARB40	Javiana	Human	USA	
SARA41	Paratyphi B	Human	France	1976		SARB41	Javiana	Human	USA	
SARA42	Paratyphi B	Human	UK	1974		SARB42	Paratyphi A			
SARA43	Paratyphi B	Human		1982		SARB43	Paratyphi B	Human	France	1976
SARA44	Paratyphi B	Human		1965		SARB44	Paratyphi B var. Java	Food	Middle East	1976
SARA45	Paratyphi B	Cow	France	1988		SARB45	Paratyphi B var. Java	Human	France	1976
SARA46	Paratyphi B	Human		1981		SARB46	Paratyphi B monophasic	Water	UK	1965
SARA47	Paratyphi B var. Java	Sewage	UK	1983		SARB47	Limete	Human	Africa	1881
SARA48	Paratyphi B var. Java	Human	UK	1982		SARB48	Paratyphi C		France	
SARA49	Paratyphi B var. Java	Sewage	UK	1982		SARB49	Paratyphi C	Human	France	1988
SARA50	Paratyphi B var. Java	Food		1976		SARB50	Oranienburg	Human	France	1977
SARA51	Paratyphi B var. Java	Human	France	1976		SARB51	Gallinarum var. Pullorum		Germany	
SARA52	Paratyphi B var. Java	Cow	France	1987		SARB52	Gallinarum var. Pullorum		Germany	
SARA53	Paratyphi B var. Java	Human	France	1988		SARB53	Reading		UK	1988
SARA54	Paratyphi B var. Java	Human	France	1988		SARB54	Rubislaw			
SARA55	Paratyphi B var. Java	Human	France	1981		SARB55	Saintpaul		USA	
SARA56	Paratyphi B var. Java	Human	France	1976		SARB56	Saintpaul		USA	
SARA57	Paratyphi B monophasic	Water	UK	1965		SARB57	Schwarzengrund		UK	1988
SARA58	Paratyphi B var. Java monophasic	Human	France	1981		SARB58	Sendai		UK	1974
SARA59	Paratyphi B var. Java monophasic	Human	France	1981		SARB59	Senftenberg	Chicken	USA	1987
SARA60	Paratyphi B var. Java monophasic	Food	France	1976		SARB60	Stanley		UK	1988
SARA61	Agona	Water	UK	1974		SARB61	Stanleyville		UK	1988
SARA62	Limete	Human		1981		SARB62	Thompson	Human	USA	
SARA63	Muenchen	Human	France	1988		SARB63	Typh	Human	Senegal	1988
SARA64	Muenchen	Cow	USA			SARB64	Typh	Human	Senegal	1988
SARA65	Muenchen	Chicken	USA			SARB65	Typhimurium	Human	Mexico	
SARA66	Muenchen	Human	USA			SARB66	Typhimurium	Parrot	USA	1987
SARA67	Muenchen	Human	Mexico			SARB67	Typhimurium		France	
SARA68	Muenchen	Human	France	1988		SARB68	Typhimurium	Heron	USA	
SARA69	Muenchen	Human	France	1988		SARB69	Typhisuis	Pig	USA	1968
SARA70	Muenchen	Human	France	1988		SARB70	Decatur	Pig	USA	1967
SARA71	Muenchen	Human	USA			SARB71	Wien		France	1988
SARA72	Muenchen	Human	France	1988		SARB72	Wien		France	1988

SARA37	Heidelberg	Turkey	USA	1987		SARB37	Newport	Human	Mexico	
SARA38	Heidelberg	Turkey	USA	1987		SARB38	Newport	Snake	USA	1987
SARA39	Heidelberg	Human	USA			SARB39	Panama		Italy	
SARA40	Heidelberg		USA			SARB40	Javiana	Human	USA	
SARA41	Paratyphi B	Human	France	1976		SARB41	Javiana	Human	USA	
SARA42	Paratyphi B	Human	UK	1974		SARB42	Paratyphi A			
SARA43	Paratyphi B	Human		1982		SARB43	Paratyphi B	Human	France	1976
SARA44	Paratyphi B	Human		1965		SARB44	Paratyphi B var. Java	Food	Middle East	1976
SARA45	Paratyphi B	Cow	France	1988		SARB45	Paratyphi B var. Java	Human	France	1976
SARA46	Paratyphi B	Human		1981		SARB46	Paratyphi B monophasic	Water	UK	1965
SARA47	Paratyphi B var. Java	Sewage	UK	1983		SARB47	Limete	Human	Africa	1881
SARA48	Paratyphi B var. Java	Human	UK	1982		SARB48	Paratyphi C		France	
SARA49	Paratyphi B var. Java	Sewage	UK	1982		SARB49	Paratyphi C	Human	France	1988
SARA50	Paratyphi B var. Java	Food		1976		SARB50	Oranienburg	Human	France	1977
SARA51	Paratyphi B var. Java	Human	France	1976		SARB51	Gallinarum var. Pullorum		Germany	
SARA52	Paratyphi B var. Java	Cow	France	1987		SARB52	Gallinarum var. Pullorum		Germany	
SARA53	Paratyphi B var. Java	Human	France	1988		SARB53	Reading		UK	1988
SARA54	Paratyphi B var. Java	Human	France	1988		SARB54	Rubislaw			
SARA55	Paratyphi B var. Java	Human	France	1981		SARB55	Saintpaul		USA	
SARA56	Paratyphi B var. Java	Human	France	1976		SARB56	Saintpaul		USA	
SARA57	Paratyphi B monophasic	Water	UK	1965		SARB57	Schwarzengrund		UK	1988
SARA58	Paratyphi B var. Java monophasic	Human	France	1981		SARB58	Sendai		UK	1974
SARA59	Paratyphi B var. Java monophasic	Human	France	1981		SARB59	Senftenberg	Chicken	USA	1987
SARA60	Paratyphi B var. Java monophasic	Food	France	1976		SARB60	Stanley		UK	1988
SARA61	Agona	Water	UK	1974		SARB61	Stanleyville		UK	1988
SARA62	Limete	Human		1981		SARB62	Thompson	Human	USA	
SARA63	Muenchen	Human	France	1988		SARB63	Typh	Human	Senegal	1988
SARA64	Muenchen	Cow	USA			SARB64	Typh	Human	Senegal	1988
SARA65	Muenchen	Chicken	USA			SARB65	Typhimurium	Human	Mexico	
SARA66	Muenchen	Human	USA			SARB66	Typhimurium	Parrot	USA	1987
SARA67	Muenchen	Human	Mexico			SARB67	Typhimurium		France	
SARA68	Muenchen	Human	France	1988		SARB68	Typhimurium	Heron	USA	
SARA69	Muenchen	Human	France	1988		SARB69	Typhisuis	Pig	USA	1968
SARA70	Muenchen	Human	France	1988		SARB70	Decatur	Pig	USA	1967
SARA71	Muenchen	Human	USA			SARB71	Wien		France	1988
SARA72	Muenchen	Human	France	1988		SARB72	Wien		France	1988
